# Antibiotic resistomes of healthy pig faecal metagenomes

**DOI:** 10.1099/mgen.0.000272

**Published:** 2019-05-15

**Authors:** Aoife Joyce, Charley G. P. McCarthy, Sinead Murphy, Fiona Walsh

**Affiliations:** 1 Department of Biology, Maynooth University, Maynooth, Co. Kildare, Ireland

**Keywords:** metagenome, healthy, pig, antibiotic resistance, microbiome, KEGG

## Abstract

Antibiotic resistance reservoirs within food-producing animals are thought to be a risk to animal and human health. This study describes the minimum natural resistome of pig faeces as the bacteria are under no direct antibiotic selective pressure. The faecal resistome of 257 different genes comprised 56 core and 201 accessory resistance genes. The genes present at the highest relative abundances across all samples were *tetW*, *tetQ*, *tet44*, *tet37*, *tet40*, *mefA*, *aadE*, *ant(9)−1*, *ermB* and *cfxA2*. This study characterized the baseline resistome, the microbiome composition and the metabolic components described by the Kyoto Encyclopedia of Genes and Genomes (KEGG) pathways in healthy pig faeces, without antibiotic selective pressures. The microbiome hierarchical analysis resulted in a cluster tree with a highly similar pattern to that of the accessory resistome cluster tree. Functional capacity profiling identified genes associated with horizontal gene transfer. We identified a statistically significant positive correlation between the total antibiotic resistome and suggested indicator genes, which agree with using these genes as indicators of the total resistomes. The correlation between total resistome and total microbiome in this study was positive and statistically significant. Therefore, the microbiome composition influenced the resistome composition. This study identified a core and accessory resistome present in a cohort of healthy pigs, in the same conditions without antibiotics. It highlights the presence of antibiotic resistance in the absence of antibiotic selective pressure and the variability between animals even under the same housing, food and living conditions. Antibiotic resistance will remain in the healthy pig gut even when antibiotics are not used. Therefore, the risk of antibiotic resistance transfer from animal faeces to human pathogens or the environment will remain in the absence of antibiotics.

## Data Summary

The sequences have been deposited in the European Nucleotide Archive (ENA) repository under the accession number PRJEB23112 and secondary accession ERP104845 and in EBI metagenomics project codes ERP104845 and PRJEB23112. These are open access data repositories. This also available on NCBI under Bioproject accession PRJEB23112 and ID 485570 and author Maynooth University.

**Impact Statement**

Antibiotic resistance may move between bacteria within the gut of animals and then be transferred to other bacteria that may cause infections in animals or humans. The aim of this work was to identify what antibiotic resistance genes are present in the faeces of pigs even when they have not been given antibiotics. We identified many genes that cause antibiotic resistance. Some of these were present in all faecal samples of the pigs and some were present in a small number. The significance of these findings is that using this information we know that if we find, e.g. *ermB* gene in a pig faecal sample that this is not unusual as it was in all our samples. However, if we find a *qnrB* gene, this is unusual, and something has happened in the gut of this pig to allow this gene to move into the bacteria present in the pig faeces. Why do we need to know this? We need to be able to see what genes move in order to identify what genes will potentially move to bacteria that cause infections in animals and humans.

## Introduction

The concept to date is that animals, in particular food animals, are potential reservoirs of antimicrobial resistance genes (ARGs) and transfer and survive in the gut microflora and transfer to human or animal pathogens [1]. In addition, the animal wastes, which are spread on agricultural land may cause additional routes of transfer of ARGs to humans via the food chain and water cycle [1]. Therefore, animals, especially animal faeces are of concern for human and environmental health. The use of antibiotics in animal husbandry and welfare have been linked to increasing antimicrobial resistance in animals and humans [2]. Antibiotics are used prophylactically at specific stages of pig growth, predominantly in order to treat diarrhea, which is especially common during the weaning stages in piglets [3].

There is a rich literature on the topic of the presence of specific ARGs, in faecal samples from animals and in environmental reservoirs such as soil or water. The ability to detect a wide array of ARGs has evolved in line with the evolution of microbiology from culture-based studies to PCR or qPCR-based and now metagenomic-based studies. Many studies of the animal faecal resistome have focused on the presence of ARGs or antibiotic-resistant bacteria present in manure [1, 4, 5]. These studies have detected a range of ARGs conferring resistance to all classes of antimicrobials. The initial studies investigated AMR in zoonotic pathogens but with the development of further molecular tools it was possible to analyse the presence of the ARGs within animal faecal or manure samples [1, 6, 7]. The advance of metagenomic analysis has enabled the detection and relative quantification of ARGs present in animal faeces such as pigs, chickens and cattle [8–11]. In addition, we can now compare the microbiome and resistome within the same samples. However, the number of studies is relatively low in comparison to that in humans. The characterization of antimicrobial resistance in environmental samples such as soil or water was technically challenging, but the use of molecular biological tools such as PCR or qPCR enabled the investigation and detection of reservoirs of ARGs in soil, water and anthropogenically amended environments [12]. The advent of metagenomic analysis has now also enabled the detection of a wide range of ARGs and the microbiome content of environmental samples of soil and water [13, 14]. The great advantage of using the same tools across different matrices is the ability to compare the data. When analysing AMR from a One Health perspective it is necessary to detect, quantify and compare AMR data across human, animal and environmental biomes. Metagenomic data may be used for this purpose. However, remaining gaps in our current tools, cost, skills and lack of standardization are still prohibitory to the routine surveillance of AMR in these biomes for many researchers and government organizations [15].

In order to analyse the risk of ARG transfer or create surveillance data across countries, techniques such as PCR can be used, which is a cost-effective tool available to all. However, using PCR to analyse samples for all known ARGs would be costly and time-consuming. Therefore, a subset of indicator ARGs must be selected to identify the spread of antibiotic resistance through the food chain and across the globe. In order to select these ARGs it is essential that the baseline antibiotic resistome in healthy animals is known. One of the initial goals of the Human Microbiome Project was to characterize the healthy human microbiome as a baseline for reference and comparison studies [16]. This study aimed to firstly identify the baseline ARGs, the microbiome composition and the metabolic components described by the Kyoto Encyclopedia of Genes and Genomes (KEGG) pathways in healthy pig faeces, without antibiotic selective pressures. Secondly, we aimed to identify if there were correlations between the resistome and microbiome. Thirdly, we wanted to use the metagenomic dataset generated to test the ability of the smaller sets of resistance genes suggested by Bengtsson-Palme to accurately rank environmental samples in terms of total (characterized and uncharacterized) resistance gene abundance and diversity [17].

## Methods

### Sample collection and DNA extraction

Fresh faeces were sampled at the rectum of 16 large white sows from the same farm. The samples were taken from each animal at the same time on one day. The animals were housed at the University College Dublin farm under the same housing, feed and environmental conditions. They had not been given antibiotics since weaning. Antibiotics must be prescribed by a veterinary practitioner in Ireland and no antibiotics were prescribed. The samples were transported immediately to the laboratory and stored at −80 °C. DNA was extracted from each sample in triplicate using the PowerSoil DNA extraction kit (Qiagen, Crawley, UK). The DNA concentrations were measured using NanoDrop technology and the Qubit dsDNA High Sensitivity Assay Kit (ThermoFisher Scientific, Dublin, Ireland).

### DNA sequencing

#### Metagenome sequencing

The final DNA concentrations were 5 ng ul^−^^1^ in 100 ul. The sequencing was performed at the Illumina approved service provider University of Liverpool Centre for Genomics Research. The samples were sequenced in Illumina HiSeq 4000 paired end sequencing (2×150 bp). We received between 60 and 101 million raw reads per sample. The raw Fastq files were trimmed for the presence of Illumina adapter sequences using Cutadapt version 1.2.1 [18]. The option -O 3 was used, so the 3′ end of any reads, which match the adapter sequence for 3 bp or more were trimmed. The reads were further trimmed using Sickle version 1.200 with a minimum window quality score of 20. Reads shorter than 10 bp after trimming were removed.

### Bioinformatics analysis

The trimmed, quality control approved reads were uploaded to the European nucleotide Archive (ENA). The uploaded files in ENA were transferred to EBI metagenomics (currently MGnify) for analysis of the metagenomic data [19]. The silva SSU/LSU version 128 was used by MGnify to assign the eukaryotic as well as prokaryotic taxa [19]. The resulting classification system was compared to QIIME/Greengenes and benchmarked using both mock community and real-world datasets to confirm validity of results [19].

### Antibiotic resistance gene analysis

The resistome profiles of each metagenomic dataset were determined using the two-stage ARGs-OAP pipeline, with all parameters as default for the pipeline [20, 21]. All paired-end reads from each dataset were initially screened for potential ARG sequences by searching against the Structured ARG database (SARG) using ublast [22]. The SARG comprises both the ARDB and CARD [22]. The microbial community within each dataset was determined by identifying 16S rRNA hypervariable regions using ublast, and copy number correction was performed for each dataset with the Copyrighter database [23]. Each set of potential ARGs per dataset were then annotated and classified using blastx on the ARGs-OAP Galaxy platform [20]. ARG types/subtypes were identified and their abundances were calculated with normalization by the 16S rRNA gene sequence length, the number of 16S rRNA genes and the ARG reference sequence length. ARG abundance was expressed as ‘copy of ARG per copy of 16S-rRNA gene’ (thereafter called ‘ratio’).

### Core vs accessory resistome assignment

The ARGs were assigned to the core resistome if the gene was present in all 16 samples. If a resistance gene was detected in one sample but less than 15 samples it was assigned to the accessory resistome.

### Statistical and data analysis

The statistical analysis of the ARGs and correlation analysis of the ARGs was performed using the PAleontological STatistics version 3.2 (past) software [24]. Using ANOVA Mann–Whitney pairwise tests were performed for between-group comparisons with Bonferroni correction for multiple comparisons separately for the datasets' core ARGs and accessory ARGs. Mann–Whitney pairwise test *P*-values are given for all Np=G(G-1)/2 pairs of groups. The asymptotic approximation described under the Mann–Whitney module is used [24].

1. Raw *P*-values, uncorrected significance: the *P*-values from each individual pairwise test, marked in yellow if *P*<0.05, not corrected for multiple testing.
2. Bonferroni corrected *P*-values: the values shown are *P*’=pNp. Marked as significant if *P*’<0.05.

The Bonferroni corrected *P*-values were used when describing significance in the results and discussion. The raw *P*-values and Bonferroni corrected *P*-values of the statistically significant differences in the core or accessory resistomes of each sample compared with every other sample are described in the Supplementary Excel File (available in the online version of this article).

The Mantel test was performed within past using the Bray–Curtis similarity index. The hierarchical cluster analysis of the microbiome OTUs was performed using Calypso version 8.56 software [25].

### Data availability

The sequences have been deposited in the European Nucleotide Archive (ENA) under primary accession PRJEB23112 and secondary accession ERP104845 and in EBI metagenomics project codes ERP104845 and PRJEB23112.

## Results

Total DNA from faecal samples from 16 pigs were analysed using shotgun sequencing. The total reads per sample analysed (after quality control and trimming) ranged from 27 343 532 reads to 39 515 474 reads.

### Antibiotic resistomes

**Fig. 1.**
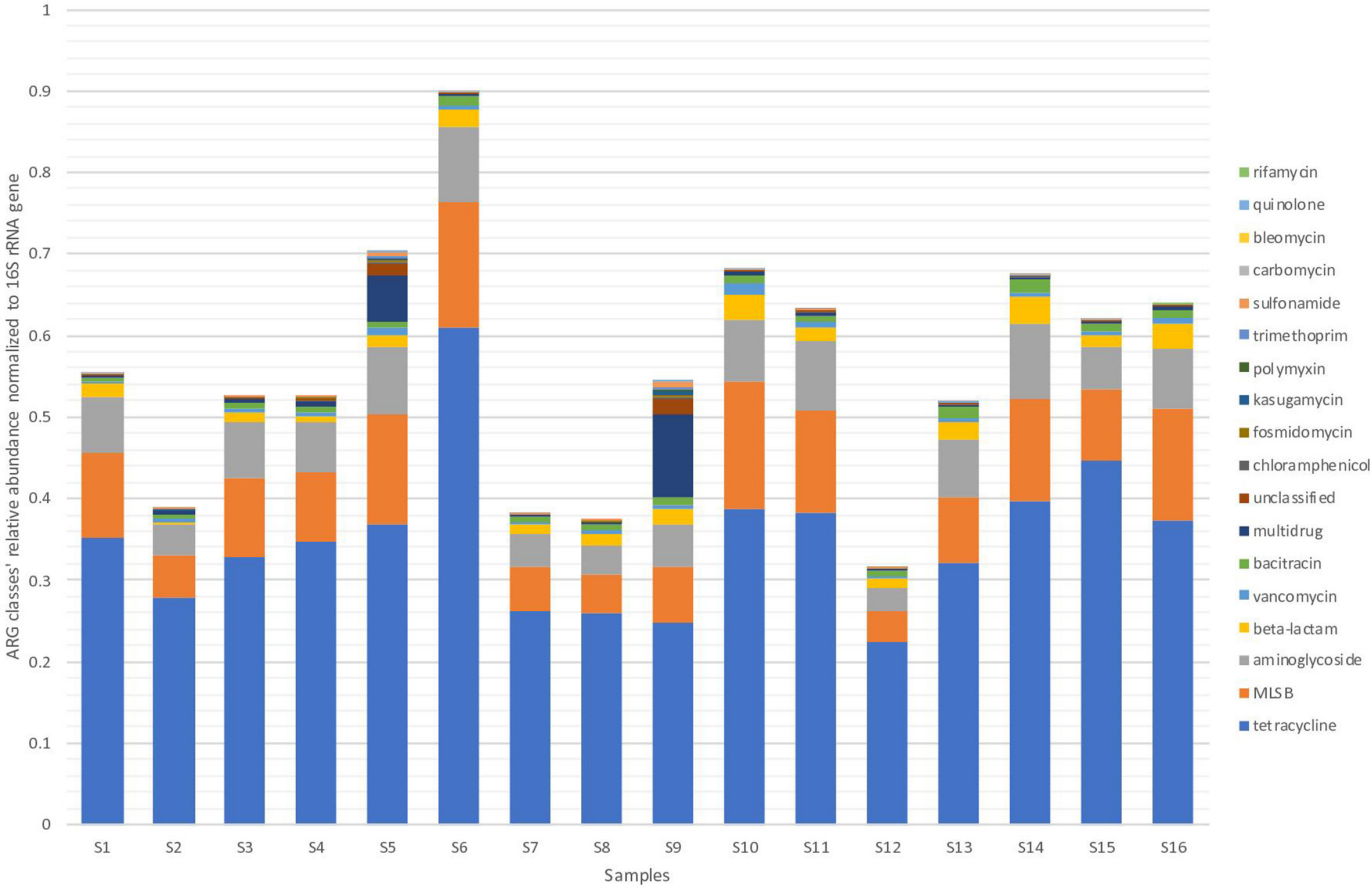
Relative abundance of ARGs per sample by antibiotic class.

In total 257 ARGs were detected across all pig faecal samples. Samples 5 and 9 contained the highest variety of ARGs (*n*=187 and 196, respectively). The relative abundance of ARGs per sample by antibiotic class were summed in order to investigate if the trends of antibiotic resistance were consistent across all samples (Fig. 1). All samples contained resistance genes from the same classes of antibiotics. Tetracycline resistance comprised the greatest proportions of ARGs present in all samples, followed by the macrolide, lincosamide, streptogramin B class (MLS_B_), aminoglycoside and beta-lactam classes. Resistance to the remaining classes of antibiotics was relatively low. The relative abundances of the total classes and specific classes varied across the samples. Sample 6 contained double the relative abundance of total resistance genes compared with samples 2, 7, 8 and 12. This was due to the presence of double the relative abundances of tetracycline resistance genes – *tetQ* and *tetL* – in comparison with the other samples. Samples 5 and 9 contained tenfold more genes classified as multi-drug resistance than the other samples.

The resistome consisted of the core resistome genes, which were present in all samples (*n*=56) and the accessory resistome genes (*n*=201) that were present in at least one but less than 16 pigs. The core resistome genes comprised aminoglycoside (*n*=5), bacitracin (*n*=2), beta-lactam (*n*=5), fosmidomycin (*n*=1), MLS_B_ (*n*=10), tetracycline (*n*=14), unclassified (*n*=2), vancomycin (*n*=8) and multi-drug resistance (*n*=9) genes. The multi-drug resistance genes were all efflux-associated genes. The tetracycline resistance genes were the largest group of genes in the core resistome. Of the five beta-lactam resistance genes detected three are not ARGs, but are enzymes involved in peptidoglycan biosynthesis [penicillin-binding proteins (PBPs)], the remaining two genes were *bla*_CFXA2_ and *bla*_CFXA3_.

### Relative abundances of the core resistome

**Fig. 2.**
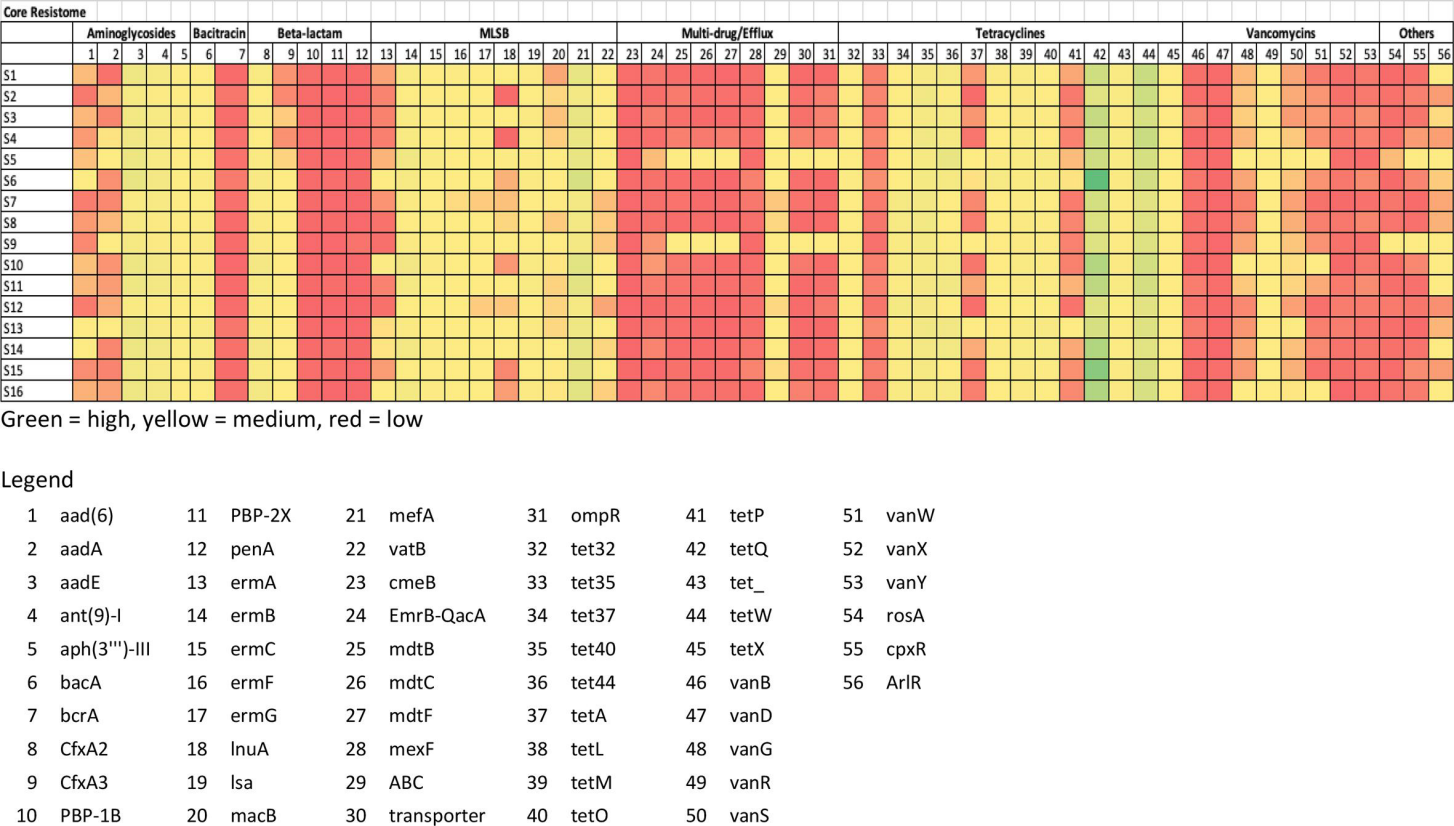
Relative abundances (copy of ARG per copy of 16S rRNA gene) of core resistome across 16 pig faecal samples.

**Fig. 3.**
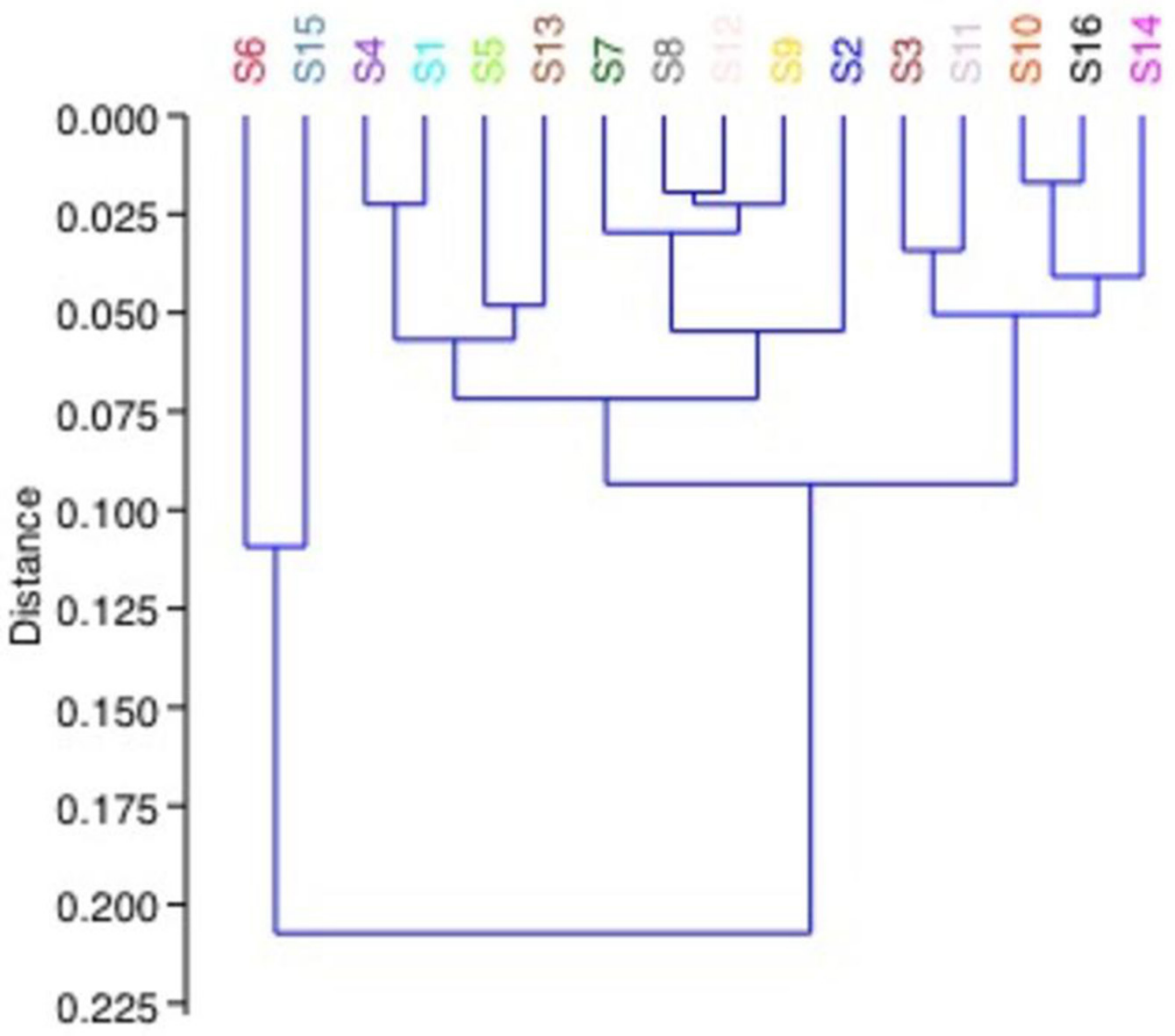
Similarity and distance matrices of the total core resistomes using Bray–Curtis.

**Fig. 4.**
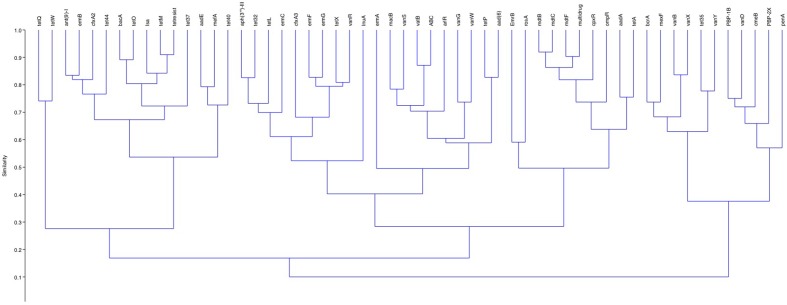
Similarity and distance matrices of the individual ARGs within the core resistomes across all samples using Bray–Curtis.

The core resistome contained 56 different resistance genes. The analysis of the data indicated that while these genes were present in all samples the relative abundances of the genes varied greatly across samples (Fig. 2). The genes present at the highest relative abundances across all samples were *tetW*, *tetQ*, *tet44*, *tet37*, *tet40*, *mefA*, *aadE*, *ant(9)−1*, *ermB* and *cfxA2*. The similarity and distance matrices using Bray–Curtis grouped the samples based on the relative abundances of their core resistomes (Fig. 3). Samples 6 and 15 clustered together but away from the remaining samples. Both samples had elevated relative abundances of the *tetQ* gene. While sample 6 had a higher total abundance of the core genes, sample 15 was similar to the other samples. The core genes *tetQ* and *tetW* clustered together and away from all other genes based on similarity and distance matrices using Bray–Curtis (Fig. 4). In addition, the multi-drug resistance genes (*mdt* genes) and the PBPs, which are chromosomal genes rather than ARGs, clustered together and apart from most other genes.

### Accessory resistome

The accessory resistome comprised of 201 genes that were detected in at least one sample but not present in all samples (Table S2). Classes to which antibiotic resistance genes were present only in the accessory resistome were sulfonamides, trimethoprim, quinolone and chloramphenicol antibiotics. The *sul1* and *sul2* genes were detected in 15 of 16 samples analysed. Each sample contained between three and eight different chloramphenicol resistance genes. Seven different *dfrA* trimethoprim resistance genes were detected across the samples. Both trimethoprim and quinolones are synthetic antibiotics and ARGs to these classes were detected in at least one sample. The *qnrB* gene was detected in samples 5 and 9 and the *qep* resistance genes in sample 13.

**Fig. 5.**
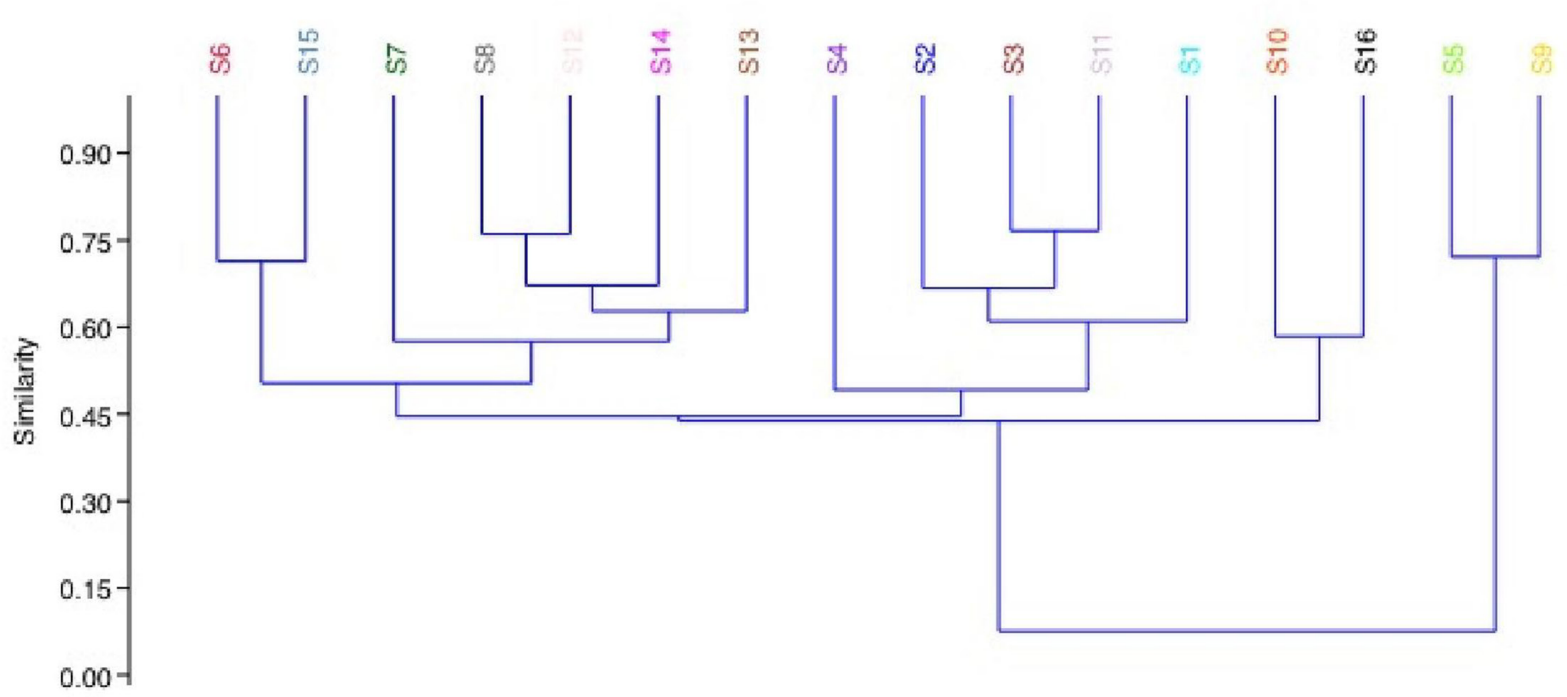
Sample similarity and distance matrices using Bray–Curtis of the relative abundances of accessory resistomes.

The cluster analysis of accessory resistomes grouped samples 5 and 9 together but separate from the remaining samples (Fig. 5). In addition, similar to the core resistome cluster analysis, samples 6 and 15 and samples 10 and 16 were similar to each other but less similar to all other samples. Samples 8 and 12 also remain closely clustered but their nearest neighbours have changed from sample 9 to samples 13 and 14. The clustering also identified that the level of similarity decreased in the accessory resistome relative to the core resistome, indicating that the variability across samples is greater in the accessory resistome.

**Fig. 6.**
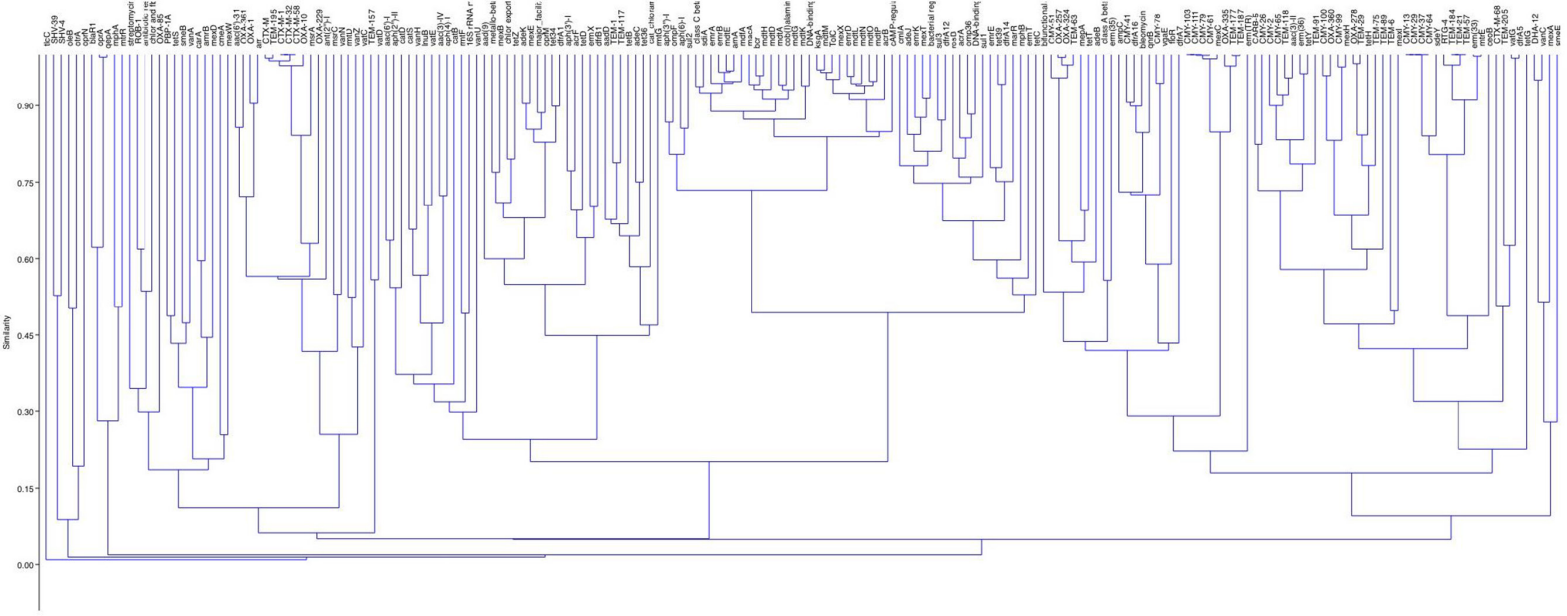
Cluster analysis of the accessory resistome.

The cluster analysis identified the rarely detected ARGs, which are distantly related to the remaining ARGs on the basis of presence in the samples, e.g. *qepA*. The *qepA* gene clustered closely with the *oprA* gene but both of these were distant to the remaining genes. The *qnrB* gene was clustered with three ARGs: a bleomycin resistance gene, *dfrA16* and *bla*_CMY-41_. Similar to the core resistome several of the multi-drug resistance genes (e.g. *mdt* genes) and the efflux genes (*tolC*, *acrB*), which are chromosomally associated, clustered together and apart from most other genes (Fig. 6).

There were no statistically significant differences between the relative abundances of the core resistomes. The accessory resistomes of both samples 5 and 9 were statistically significantly different to all other samples except each other (Supplementary Excel File). The accessory resistome of sample 16 was significantly different to all other samples except 2, 3, 4 and 11. The accessory resistome of sample 4 was significantly different to eight other samples (1, 5, 6, 7, 8, 9, 12 and 14). The specific difference in relative abundances of the ARGs can be seen in the heatmaps as a colour change (Supplementary Excel File). The core ARGs with changes comprising a tenfold difference in abundance between samples were *aad*(6), *aadA*, *cfxA3*, *ermA*, *inuA*, *macB*, *vatB*, *tetA*, *tetL*, *tetP*, *vanB*, *vanG*, *vanS*, *vanW*, *vanX*, *vanY* and *rosA* (Fig. 2).

### Microbiome analysis

**Fig. 7.**
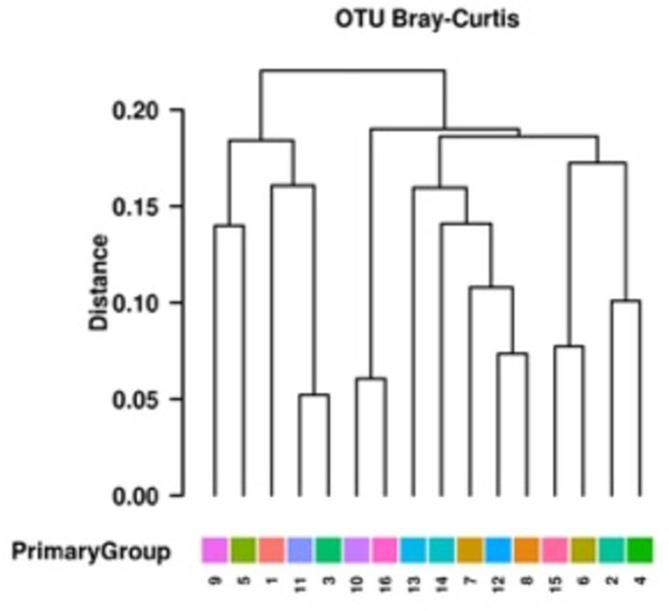
Hierarchical cluster analysis of total OTU in sample microbiomes.

The phyla that comprised the bacterial microbiome in the Irish samples were the same phyla that comprised the samples obtained by Xiao *et al*. from pig faeces obtained from farms in France, China and Denmark (Fig. S1) in terms of type and relative abundances [11]. However, the Irish samples contained a lower relative abundance of unassigned taxa. The most abundant phyla in both sets of data were Firmicutes, Bacteroietes and Tenericutes. The total OTU microbiome hierarchical analysis of the Irish pig samples resulted in a cluster tree with a highly similar pattern to that of the accessory resistome cluster tree (Fig. 7). This indicates that the microbiome composition and the accessory resistomes of the healthy pig faeces are closely linked and have a high degree of association. Findings of Munk *et al*. identified that samples with similar taxonomic compositions tended to have similar resistomes in their studies of European pig faeces [26]. However, they did not analyse if there was a distinction between the core resistomes and the accessory resistomes.

### Correlation between total resistomes and microbiomes

The correlations between total resistome and total OTUs (data from Bacterial kingdom to genera, Archaea, Eukaryotes, viruses and bacterial taxa) were analysed for the 16 samples using the Mantel test [27, 28]. Pearson’s correlation coefficient (*R*) was positive (0.3861) and there was significant positive correlation (*P*-value=0.001).

### Correlation between indicator ARGs and total resistome

Bengtsson-Palme suggested that a small sets of resistance genes may be used to accurately rank environmental samples in terms of total (characterized and uncharacterized) resistance gene abundance and diversity [17]. He described ten genes as predictors for resistance (*tetQ*, *aph3′-Ib, vanRG*, *tetV*, *dfrC*, *tetW*, *bla*_TEM_, *otrA*, *tetX*, *tetM*) [16]. The ARGs *tetQ***,***tetW*, *tetX* and *tetM* were present in the core resistome and the ARGs *aph3′-Ib, bla*_TEM_ and *otrA* were in the accessory resistomes of this study. The remainder were not detected in any sample (*vanRG*, *dfrC*, *tetV*). The Simpson diversity index is a measure of diversity, which takes into account the number of species present, as well as the relative abundance of each species in taxonomic terms. In our analysis the species are replaced by ARGs. Using the Mantel correlation between the Simpson diversity indices for the total antibiotic resistome in comparison to the genes present from the predictors ARGs (*n*=7) we identified a statistically significant (*P*=0.0005) positive correlation *R*=0.7656 between the indicator ARGs detected in our samples and the resistomes.

### Functional capacity profiling of gut microbiome

Genes with a relative abundance of greater than 0.001 % were included for further analysis. This cut-off was chosen in order to remove any potential false positives due to low sequence copy number and compare with previous studies of animal gut metagenomes that had used this cut-off [29]. A total of 1068 genes were assigned KEGG Gene Ontology (GO) terms in at least one of the 16 samples [data available on the EBI metagenomics project number ERP104845 (PRJEB23112)]. The GO terms were segregated into biological processes (*n*=411 GO terms), cellular components (*n*=51 GO terms) and molecular functions (*n*=606 GO terms).

The biological processes comprised 311 GO terms present in all samples. Almost all of these genes were involved in bacterial biological processes. However, all samples contained virion assembly genes and genes described as ‘response to antibiotics’. These animals were not treated or fed antibiotics since weaning thus the response to antibiotics is only the natural response in the microbiome of the gut. The presence of the remaining 100 genes varied in their functional identities. Fifty-five genes occurred in less than 50 % of the samples and 32 of these were present in samples 5 and 9 only. The 55 genes included genes that would not normally be associated with the gut microbiome, for example, arsinite transport (samples 5, 9), bioluminescence (samples 8, 9, 14), chitin catabolic processes (samples 3, 10, 11, 16), conjugation (sample 9), microtubule-based movement (sample 3) and response to UV (samples 5, 14).

The cellular components comprised 51 GO terms from which 36 were present in all samples and 15 were present in 14 or less samples. The cellular components with the highest presence were basic cellular components, including membrane, ribosome and cytoplasm. The presence of the septin gene and the viral capsid in all samples indicates the presence and importance of fungi and viruses as part of the pig faecal microbiome. In relation to antibiotic resistance, the presence of extrachromosomal circular DNA cellular components in all samples at relatively high gene abundance may indicate the presence of a relatively high amount of plasmid genes in all samples. However, although sample 9 contained conjugation-associated genes it did not contain a higher abundance of extrachromosomal circular DNA cellular components. Therefore, these extrachromosomal circular DNA genes may be an indication of genes associated with yeast or non-bacterial microbes [30].

The molecular function genes comprised 606 GO terms, of which 483 were present in all samples and 123 were present in 15 or less samples. The molecular functions common to all samples included ATP, DNA, RNA and protein binding, DNA replication and transport enzymes and metal-binding enzymes. The aminoglycoside 3-*N*-acetyltransferase resistance gene was detected in 12 of the samples. Beta-lactamase activity was detected only in sample 10. Both samples 5 and 9 contained the largest variety of molecular functions in the group of 123 genes, which totalled 79 and 87 genes, respectively. The remaining samples contained between 26 and 67 molecular function genes.

## Discussion

This study provided the core and accessory resistomes of the healthy pig faeces under controlled conditions, in the absence of antibiotics, which may be used as a reference set for future resistome studies. Scientists, government agencies and international organizations are currently trying to establish One Health surveillance protocols and lists of genes that may be used as indicators of antibiotic resistance pollution or transfer between animals, food, the environment and humans. The detection of the resistance genes must be translated into risk to environment or human or animal health.

There are several possible reasons for the presence of ARGs in the faecal microbiome of healthy non-antibiotic treated pigs, likewise for any animal.

### The bacteria naturally contain ARGs on their chromosomes

Some of the bacteria present in the gut as natural flora naturally harbour ARGs as part of their chromosomal DNA. Whether intrinsic resistance genes should be classified as ARGs is under debate by microbiologists. This is due to the fact that, for example, any pathogen known to harbour an AmpC will not be treated with penicillin. Others argue that these genes may move into susceptible pathogens and are therefore relevant. This study identified a core resistome in the faecal samples comprising 56 ARGs. As the microbiomes of the pigs were stable across all animals it is the core ARGs that are most likely to be the intrinsic or chromosomally mediated ARGs.

### ARGs are transferred to the gut bacteria through horizontal transfer

The ARGs most likely to be mobile are those of the accessory resistomes. For example, genes such as the plasmid-mediated quinolone resistance genes *qepA* and *qnrB* have never been reported as present on the chromosomes of the natural bacterial flora of the gut. Therefore, these are most probably imported into the gut via horizontal transfer. Whether these mobile resistance genes are transferred at or before birth or at some point during the lifetime is unknown. However, it is clear from the data that not all pigs contain the same mobile resistance genes and despite the similarities in their living conditions there is variation in their mobile resistomes.

### The presence of ARGs has been selected due to factors other than antibiotic use

Antimicrobial resistance plasmids may contain virulence genes or other genes conferring a fitness advantage to the bacteria. These bacteria and their ARGs are then selected through non-antibiotic selective pressures and the ARGs are co-selected.

### Animals are continuously with the ARGs and bacteria from their environments or food

The link between the presence of ARG and the source of ARG is very difficult in the real world. It is possible that the gut resistome is constantly seeded from external sources such as the living environment or feed sources. This would then ensure the constant provision of antimicrobial-resistant bacteria to the gut, even if the selective pressure of antimicrobials is absent.

Two previous studies measured the inter-country variations in the resistomes of pig faeces and determined the ARGs detected in several European countries and China [11, 26]. These studies used pig farms with varying levels of antibiotic use. The study of the Xiao *et al*. identified that ARGs with the highest prevalence were those associated with resistance to bacitracin, cephalosporin, macrolide, streptogramin B or tetracycline, and therefore could be described as the core resistome [11]. The additional core ARGs to chloramphenicol, gentamycin B, kanamycin and neomycin were also present in all pigs but with a much lower abundance in the French and Danish pigs compared to the Chinese pigs. This study of Irish pigs identified a higher variety (*n*=56) of core ARGs. The core resistome comprised very similar families to the Xiao *et al*. study conferring resistance to the bacitracin, MLS_B_, tetracycline and aminoglycosides antibiotic classes, in addition the core resistomes of the Irish pigs contained beta-lactam, fosfidomycin and vancomycin ARGs [11]. The same chloramphenicol resistance genes were not detected in all Irish samples and therefore, were not part of the Irish core resistome, however, each sample contained at least one ARG associated with chloramphenicol resistance.

The European study of Munk *et al*. also detected a core resistome comprising 33 ARGs [26]. Eight of the ARGs that differed in abundances between countries conferred resistance to a subset of the classes of antibiotics described previously by Xiao *et al*. and in this study: chloramphenicol, macrolides, metronidazole, linezolid, tetracycline and aminoglycosides [*cat(pC194*), *ermB*, *ermF*, *inuA*, *nimJ*, *optrA*, *tet(40*) and *aac(6′)-Im*] [11]. Within these genes *cat(pC194*), *nimJ*, *optrA* and *aac(6′)-Im* were not detected in any Irish sample. The remaining genes [*ermB*, *ermF*, *inuA* and *tet(40*)] were detected in all samples and were present in the most abundant 50 % of ARGs in all samples. Similar to these two studies we also did not detect either the colistin resistance *mcr-1* or the *mcr* variant genes or the *bla*_NDM_ genes.

The Xiao *et al*. study identified fluoroquinolone resistance genes only in the Chinese samples and not in the French or Danish pig faecal samples [11]. The European study of poultry and pig faeces described the fluoroquinolone resistance genes only in the poultry samples [26]. Therefore, this study describes the first European detection of fluoroquinolone resistance genes (*qnrB* and *qep*) in pig faecal metagenomes.

The ARGs present in this study that were recommended by Bengtsson-Palme as predictors for resistance were positively correlated (statistically significant) with the total resistomes of the samples [17]. Therefore, these ARGs were predictors of resistance in the healthy pig faecal metagenomes. The correlation between total resistome and total microbiome OTUs in this study was positive and there was significant positive correlation. This has demonstrated that the microbiome composition influenced the resistome composition. However, the converse may also be true in that the microbiome could be shaped in part by the resistome present, and some organisms may not have been able to compete due to the presence of these genes or the production of products from these genes. The microbiome of environmental and animal samples has previously been shown to be significantly correlated with the antibiotic resistomes of environmental, human and animal samples [26, 31, 32]. The phylogenetic cluster tree of the genera of each sample separated samples 5 and 9 from the other samples. This was also the same in the cluster analysis of the accessory resistome. While each study has demonstrated the correlation between microbiome and resistome, it is yet to be determined which specific genera are key to the resistome or if there are clusters of genera contributing to the differing resistomes. Metagenomic and other molecular biology-based techniques have limitations and advantages over culture-based techniques in the analysis of the antibiotic resistome of agricultural ecosystems [33]. Whilst the core and accessory ARGs present in a wide global spread of pig faecal samples has now been established, techniques such as epicPCR may provide the way to identify the bacterial hosts of these ARGs across a range of hosts or environments [34].

This study identified a core and accessory resistome present in a cohort of healthy pigs. It highlights the presence of antibiotic resistance in the absence of antibiotic selective pressure and the variability between animals even under the same housing, food and living conditions. The correlation between total resistome and total microbiome in this study was positive and statistically significant. Therefore, the microbiome composition influenced the resistome composition. Using the metagenomic dataset generated we tested the ability of the smaller sets of resistance genes suggested by Bengtsson-Palme to accurately rank environmental samples in terms of total (characterized and uncharacterized) resistance gene abundance and diversity [17]. The subset of ARGs suggested by Bengtsson-Palme as potential indicator ARGs correlated well with the total metagenomic resistomes in this study [17]. In addition, we would suggest including some of the rarely found ARGs in healthy animals in Europe or the environment such as those suggested by Berendonk *et al*.[12]. This combination would enable the identification of changes in the resistome in terms of the common resistome and the emergence of problem ARGs such as *bla*_NDM_ in animals or the environment.

## Supplementary Data

Supplementary File 1

Supplementary File 2
