# Supplementary material for: Antibiotic resistomes of healthy pig faecal metagenomes
Source: Microb Genom. 2019 May 15;5(5):e000272. doi: 10.1099/mgen.0.000272 (PMC6562245; doi:10.1099/mgen.0.000272)
Supplement: Supplementary File 1 [file mgen-5-272-s001.pdf]

Supplemental Figure 1. The microbiome composition of this study samples in comparison to pig faeces obtained from farms in France, China and Denmark.

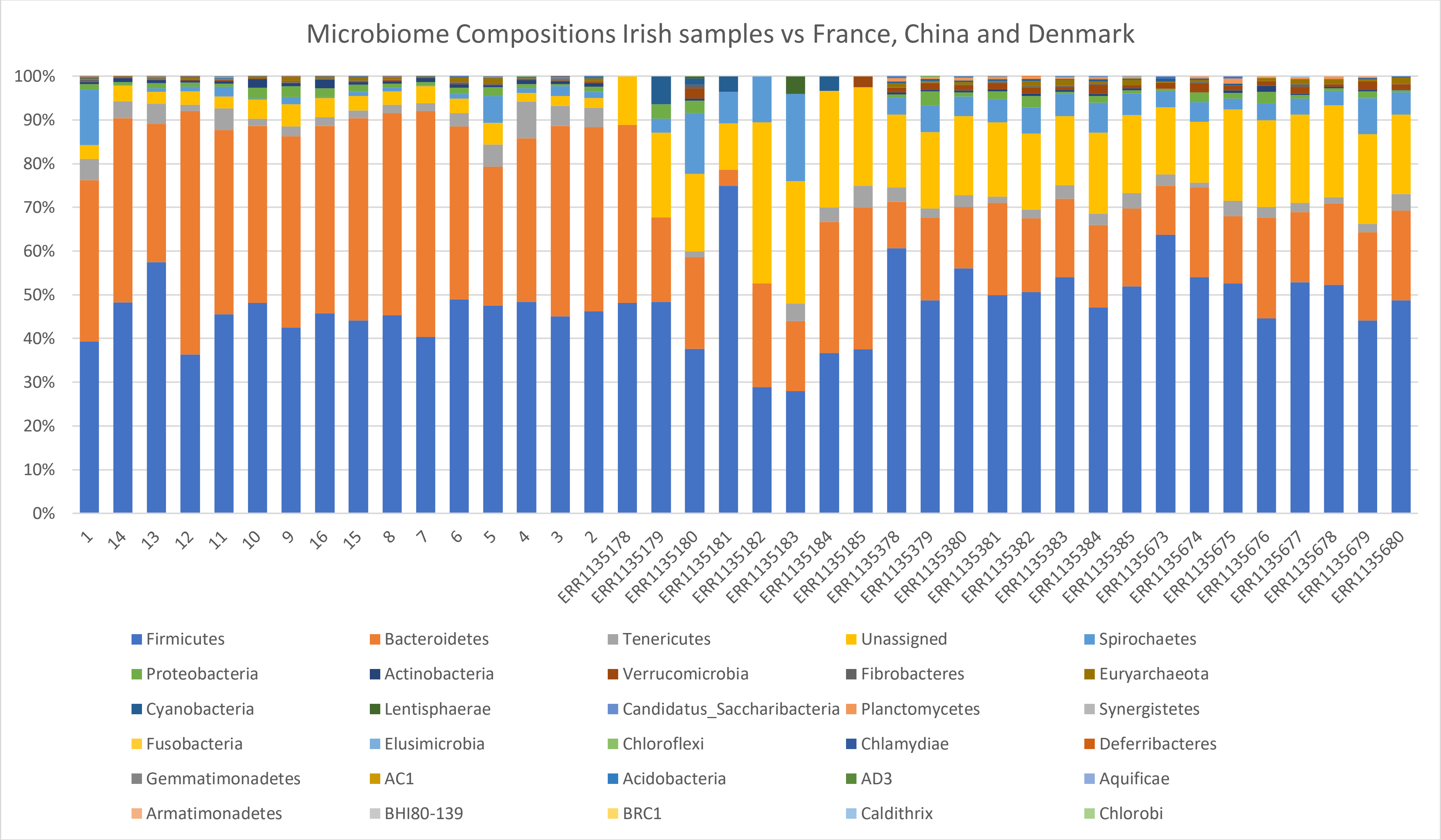


Footnote: The top 30 phyla are described in the legend.
